# Deep Learning Virtual Contrast‐Enhanced T1 Mapping for Contrast‐Free Myocardial Extracellular Volume Assessment

**DOI:** 10.1161/JAHA.124.035599

**Published:** 2024-09-30

**Authors:** Sebastian Nowak, Leon M. Bischoff, Lenhard Pennig, Kenan Kaya, Alexander Isaak, Maike Theis, Wolfgang Block, Claus C. Pieper, Daniel Kuetting, Sebastian Zimmer, Georg Nickenig, Ulrike I. Attenberger, Alois M. Sprinkart, Julian A. Luetkens

**Affiliations:** ^1^ Department of Diagnostic and Interventional Radiology University Hospital Bonn Bonn Germany; ^2^ Quantitative Imaging Laboratory Bonn (QILaB) University Hospital Bonn Bonn Germany; ^3^ Department of Internal Medicine II, Heart Center University Hospital Bonn Bonn Germany; ^4^ Department of Diagnostic and Interventional Radiology University Hospital Cologne Cologne Germany

**Keywords:** cardiovascular magnetic resonance, deep learning, extracellular volume, generative adversarial networks, T1 mapping, Machine Learning

## Abstract

**Background:**

The acquisition of contrast‐enhanced T1 maps to calculate extracellular volume (ECV) requires contrast agent administration and is time consuming. This study investigates generative adversarial networks for contrast‐free, virtual extracellular volume (vECV) by generating virtual contrast‐enhanced T1 maps.

**Methods and Results:**

This retrospective study includes 2518 registered native and contrast‐enhanced T1 maps from 1000 patients who underwent cardiovascular magnetic resonance at 1.5 Tesla. Recent hematocrit values of 123 patients (hold‐out test) and 96 patients from a different institution (external evaluation) allowed for calculation of conventional ECV. A generative adversarial network was trained to generate virtual contrast‐enhanced T1 maps from native T1 maps for vECV creation. Mean and SD of the difference per patient (ΔECV) were calculated and compared by permutation of the 2‐sided *t* test with 10 000 resamples. For ECV and vECV, differences in area under the receiver operating characteristic curve (AUC) for discriminating hold‐out test patients with normal cardiovascular magnetic resonance versus myocarditis or amyloidosis were tested with Delong's test. ECV and vECV showed a high agreement in patients with myocarditis (ΔECV: hold‐out test, 2.0%±1.5%; external evaluation, 1.9%±1.7%) and normal cardiovascular magnetic resonance (ΔECV: hold‐out test, 1.9%±1.4%; external evaluation, 1.5%±1.2%), but variations in amyloidosis were higher (ΔECV: hold‐out test, 6.2%±6.0%; external evaluation, 15.5%±6.4%). In the hold‐out test, ECV and vECV had a comparable AUC for the diagnosis of myocarditis (ECV AUC, 0.77 versus vECV AUC, 0.76; *P*=0.76) and amyloidosis (ECV AUC, 0.99 versus vECV AUC, 0.96; *P*=0.52).

**Conclusions:**

Generation of vECV on the basis of native T1 maps is feasible. Multicenter training data are required to further enhance generalizability of vECV in amyloidosis.

**Nonstandard Abbreviations and Acronyms**
CE: contrast‐enhanced
ECV: extracellular volume
GAN: generative adversarial network
GBCA: gadolinium‐based contrast agent
RFR: random forest regression
vCE: virtual contrast‐enhanced
vECV: virtual extracellular volume
ΔECV: extracellular volume difference per patient

**Clinical Perspective**

**What Is New?**

- A generative adversarial network created virtual contrast‐enhanced T1 maps for contrast‐free, virtual extracellular volume (ECV) fraction assessment. The technique of ECV estimation is used in cardiovascular magnetic resonance, especially for better characterization of inflammatory, infiltrative, and fibrotic myocardial disease.
- Virtual ECV maps had a substantial agreement with conventional ECV maps. Virtual ECV allowed the disease‐specific diagnosis of myocarditis and amyloidosis with a comparable diagnostic performance as conventional ECV.

**What Are the Clinical Implications?**

- Deep learning estimation of ECV by generating virtual contrast‐enhanced T1 maps from native T1 maps might facilitate faster and more focused cardiovascular magnetic resonance examinations without the need for gadolinium‐based contrast agents.

Cardiovascular magnetic resonance (CMR) is an important imaging modality for diagnosis and follow‐up of patients with various cardiomyopathies.^1^, ^2^ The clinical success of CMR is owed to its unique capabilities to apply different sequences for a detailed assessment of myocardial function, myocardial edema and inflammation, and myocardial fibrosis.^3^, ^4^, ^5^ Furthermore, techniques like extracellular volume (ECV) fraction mapping provide a quantitative evaluation of underlying myocardial disease including the quantification of myocardial fibrosis and inflammation.^6^

Myocardial ECV estimation exploits the nature of gadolinium‐based contrast agents (GBCAs) to accumulate in the extracellular space. In a state of dynamic equilibrium with balanced GBCA concentrations between the blood and the extracellular space, ECV can dichotomize the myocardium into an interstitial and cellular compartment. ECV can be calculated using native and contrast‐enhanced (CE) T1 mapping normalized for hematocrit.^7^ In addition to myocardial fibrosis, ECV values are increased in myocardial edema like in acute myocarditis and are considered a quantitative biomarker for the detection of diffuse disease.^4^, ^8^ ECV also enables noninvasive quantification of myocardial amyloid deposition and can influence treatment decisions in cardiac amyloidosis.^9^

In clinical routine, however, the acquisition of ECV is time consuming, and ECV map generation complicates clinical workflow due to the need of acquiring a CE T1 map in the same slice position as the native scan, the need for a peripheral venous catheter, and the need for image registration. Furthermore, the use of GBCAs might not be completely risk free, especially for patients with impaired renal function.^10^ Although the use of GBCAs is considered safe in people with preserved renal function, reports of gadolinium deposition in the brain and other organs with still not fully investigated long‐term biological effects prompts reasonable usage of GBCAs in clinical routine.^11^, ^12^

Recent studies investigated the utility of deep learning for the generation of virtual CE magnetic resonance images to increase patient safety and to decrease the economic and environmental effects of GBCA usage.^13^, ^14^, ^15^ Research on deep learning–generated CE magnetic resonance images has been mostly applied to brain imaging, and only few studies reported their potential on CMR.^13^ These studies employed a generative adversarial network (GAN) to estimate the delayed distribution of GBCAs on late gadolinium–enhanced images from native cine and T1 mapping.^16^, ^17^ In our study, we hypothesized that GANs could enable the contrast‐free generation of virtual extracellular volume (vECV) maps. Therefore, as a proof of principle, we aimed to investigate the application of GANs to generate virtual contrast‐enhanced (vCE) T1 maps from native T1 maps to assess ECV without the use of GBCA.

## Methods

### Data Set

The study complies with the principles of the Helsinki Declaration and was approved by the local institutional review board that waived informed consent due to retrospective study design (reference number: 271/23). The data that support the findings of this study are available from the corresponding author upon reasonable request. This study included pairs of native and CE myocardial T1 maps from similar clinically 1.5 Tesla CMR scanners (1.5 T Ingenia, Philips Healthcare), which were acquired in the same short‐axis orientation (basal, midventricular, and apical sections). All maps were acquired with a standard 3(3)3(3)5 modified Look‐Locker inversion‐recovery acquisition scheme before and 10 minutes after administration of a single bolus of 0.2 mmol/kg body weight of gadobutrol (Gadovist, Bayer Healthcare).^18^

Patients who underwent CMR with T1 mapping for various clinical indications between January 2019 and August 2021 at the University Hospital Bonn were consecutively identified by a radiology resident (L.M.B., 3 years of experience in CMR), who was instructed by a board‐certified cardiovascular radiologist (J.A.L, 11 years of experience in CMR). To enable a thorough evaluation of vECV estimation in relation to myocarditis and amyloidosis, for which ECV estimation is of particular interest in clinical routine, additional patients were specifically identified applying a broader time range (July 2017 to July 2023). Acute myocarditis was diagnosed on the basis of diagnostic criteria for clinically suspected myocarditis as recommended by the European Society of Cardiology Working Group on myocardial and pericardial diseases.^4^, ^19^ Cardiac amyloidosis was diagnosed on the basis of myocardial biopsy, elevated light‐chain immunoglobins, technetium‐99m or 3,3‐diphosphono‐1,2‐propanodicarboxylic acid scintigraphy or biopsy of another involved organ.^20^

CMR diagnosis and, if available, a hematocrit value within 48 hours before CMR were extracted from the clinical information system. Patients without available hematocrit were used to train and validate the GAN to generate vCE T1 maps using 5‐fold cross‐validation. Patients with available hematocrit enabling calculation of reference conventional ECV were included in a hold‐out test set to investigate the utility of vCE for contrast‐free vECV estimation.

Additionally, to create an external evaluation cohort, a radiology resident (K.K., 5 years of experience in CMR, supervised by L.P., a board‐certified cardiovascular radiologist with 8 years of experience in CMR) specifically identified patients with CMR and recent hematocrit value of another institution (University Hospital of Cologne) between January 2021 and May 2024. External validation CMR was conducted at 1.5 T (Ingenia, Philips Healthcare) with administration of 0.2 mmol/kg body weight of gadobutrol but with a different 5(3)3 modified Look‐Locker inversion‐recovery acquisition scheme. Data S1 describes in detail the data preprocessed before training the deep learning method consisting of myocardial segmentation, rigid registration, cropping, and T1 value normalization. T1 map pairs with poor image quality (ie, due to severe motion or banding artifacts) or with failed rigid registration (due to obvious dissimilar orientation or cardiac phase) were excluded from analysis.

### Generative Adversarial Network Training for vCE T1 Maps

As generator for vCE T1 maps, we employed a U‐Net–like convolutional neural network, which is widely applied for medical image segmentation, but also for deep learning–based generation of CE magnetic resonance images.^14^, ^21^, ^22^, ^23^, ^24^, ^25^ We applied 3 common loss functions also used in previous studies to optimize the U‐Net generator:

- Pixel‐wise mean absolute distance loss^26^
- Structural similarity index loss^27^
- Adversarial loss via “PatchGAN” discriminator^26^

**Figure 1:**
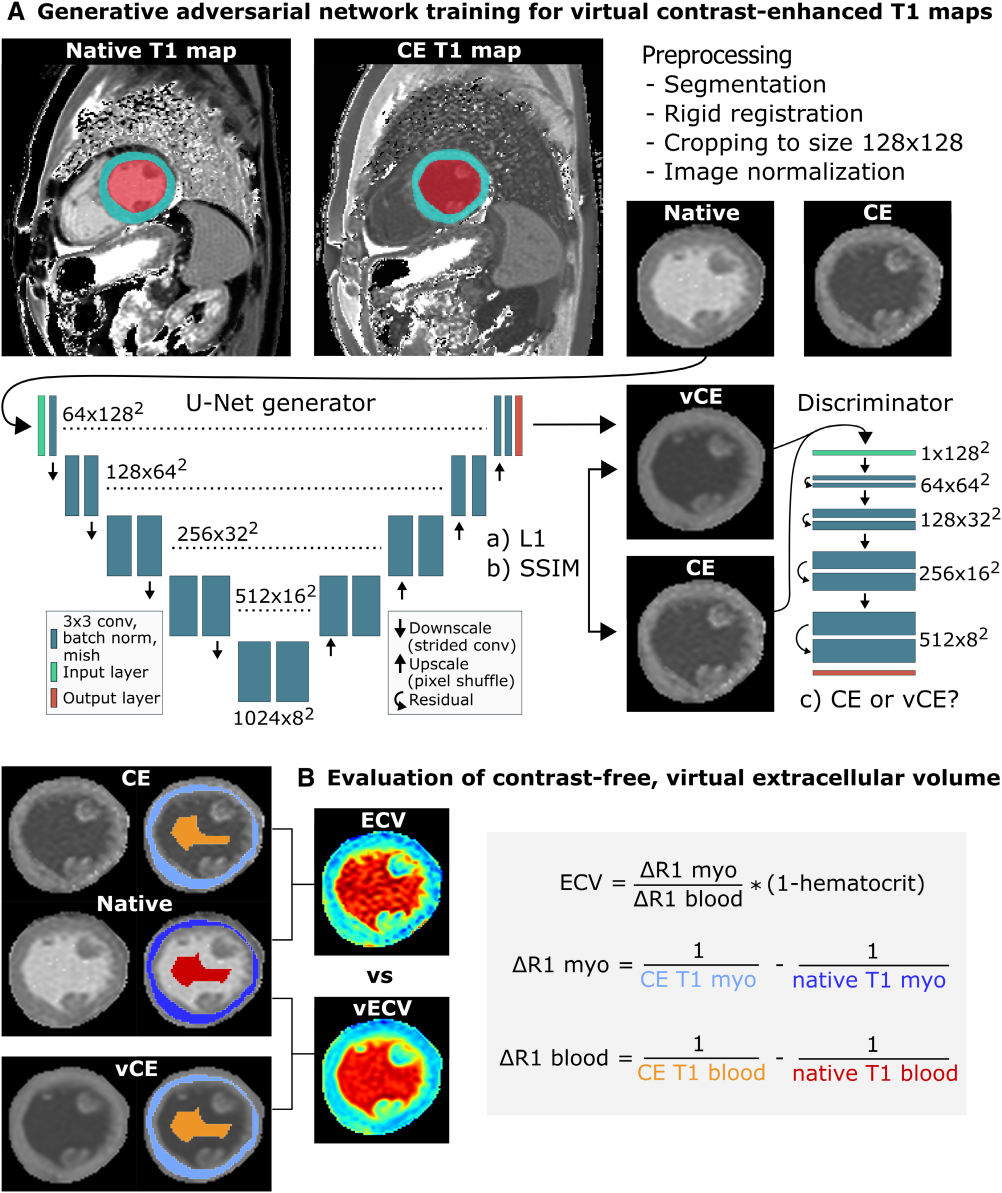
Overview of the presented approach for generating contrast‐free, virtual extracellular volume (vECV). **A**, Illustration of the generative adversarial network (GAN) used to generate virtual contrast‐enhanced (vCE) T1 maps that were used to calculate vECV fraction. The GAN consists of a generator that learns to generate the vCE maps by comparing its predictions with the conventional contrast‐enhanced (CE) T1 map and by deceiving a discriminator into recognizing the vCE maps as conventional CE. The GAN model was trained with 5‐fold cross‐validation on the developmental data set without current hematocrit. **B**, The trained model was applied to the hold‐out test data set to generate vCE T1 maps. Myocardial (myo) and blood ΔR1 values were derived from native T1 maps in combination with conventional CE T1 and with vCE T1 maps to calculate conventional extracellular volume (ECV) and virtual extracellular volume fraction (vECV).

Figure 1 illustrates the investigated deep learning architecture and losses in detail. Detailed information on hyperparameters used during training can be found in Data S2. The methods for vCE T1 map generation were trained with 5‐fold cross‐validation on T1 maps from the University Hospital Bonn. We compared the GAN‐based approach with a random forest regression (RFR). As with the GAN, we developed the RFR on the training set (without hematocrit) to predict mean vCE T1 values of myocardium and of the blood pool on the basis of the mean native T1 values of myocardium and the blood pool. In contrast to the GAN, we additionally included the age and sex of the patient as input variables for the RFR. RFR was implemented using Scikit‐learn version 1.1.2 using default parameters (Data S3).

### Evaluation of Contrast‐Free, vECV Estimation

For the hold‐out test set with recent hematocrit allowing for calculation of ECV, all 5 cross‐validated GAN models were applied in an ensemble for generation of vCE T1 maps by pixel‐wise aggregation of the maps from each model. The generated vCE T1 maps were evaluated for calculating contrast‐free vECV. Myocardial ECV/vECV fraction was calculated using mean native and CE/vCE T1 map values within a region of interest in the myocardium and a region of interest within blood. Detailed information on region of interest creation can be found in the Data S4. Figure 1 illustrates the ECV calculation and the applied formula.^28^ For visual assessment of vECV, patients with visible focal or diffuse lesions were identified on the conventional ECV and vECV map by a board‐certified cardiovascular radiologist (J.A.L.). Lesions were further classified as follows: (A) lesions present in the same location in the conventional ECV and vECV map, (B) lesions present in different locations in vECV compared with conventional ECV, (C) no lesions present in vECV but in conventional ECV, and (D) lesion in the vECV map but no lesion in the conventional ECV map.

### Statistical Analysis

Statistical analysis was conducted in Python version 3.9.12 (Python Software Foundation, Beaverton, OR) using SciPy version 1.9.0, Scikit‐learn version 1.1.2, statsmodels version 0.13.2 and pROC version 1.18.5 for R, executed in Python by r2py version 3.5.14.^29^, ^30^, ^31^, ^32^ Separated into different subgroups on the basis of primary CMR diagnosis, differences between CE and vCE T1 on the validation data were tested by permutation of *t* test comparing mean of the difference per patient (ΔCE T1). Also, separated into different subgroups on the basis of CMR diagnosis, differences on hold‐out test set and on the external validation cohort with recent hematocrit between conventional ECV and vECV was also tested using a permutation of the *t* test and by comparing mean of the difference per patient (ΔECV) for the GAN and RFR models. For ΔCE T1 and ΔECV, SDs are also reported. The *t* test was applied only if the group size was ≥5. For the hold‐out test set, correlation between conventional ECV and GAN‐based vECV was assessed by linear regression analysis with the Pearson correlation coefficient (*r*) and systematic differences were investigated using Bland–Altman analysis.

A previously published ECV cutoff of our group derived from ECV maps of myocarditis cases of the University Hospital Bonn of 28.8% was applied for both conventional ECV and vECV of the hold‐out test set to differentiate between patients with disease and patients with normal CMR.^4^, ^5^ Diagnostic performance to differentiate between patients with normal CMR and patients with acute myocarditis or amyloidosis was assessed by area under the receiver operating characteristic curve (AUC). AUC differences between conventional ECV and vECV were tested with Delong's test. Sensitivity and specificity were determined. Differences between ECV and vECV were also tested by McNemar's test. Two‐sided permutation tests with 10 000 resamples and the permutation type “samples” were applied for McNemar's and *t* tests using SciPy. For Delong's test, a 2‐sided permutation version with 10 000 resamples using the method described by Venkatraman and Begg were conducted using pROC in R (R Foundation for Statistical Computing, Vienna, Austria).^33^ Ninety‐five percent CIs were determined by bootstrapping with 10 000 resamples for AUC, sensitivity and specificity, and for the difference between conventional ECV and vECV in these metrics. *P*<0.05 was considered indicative of a statistically significant difference.

## Results

### Data Set

**Figure 2:**
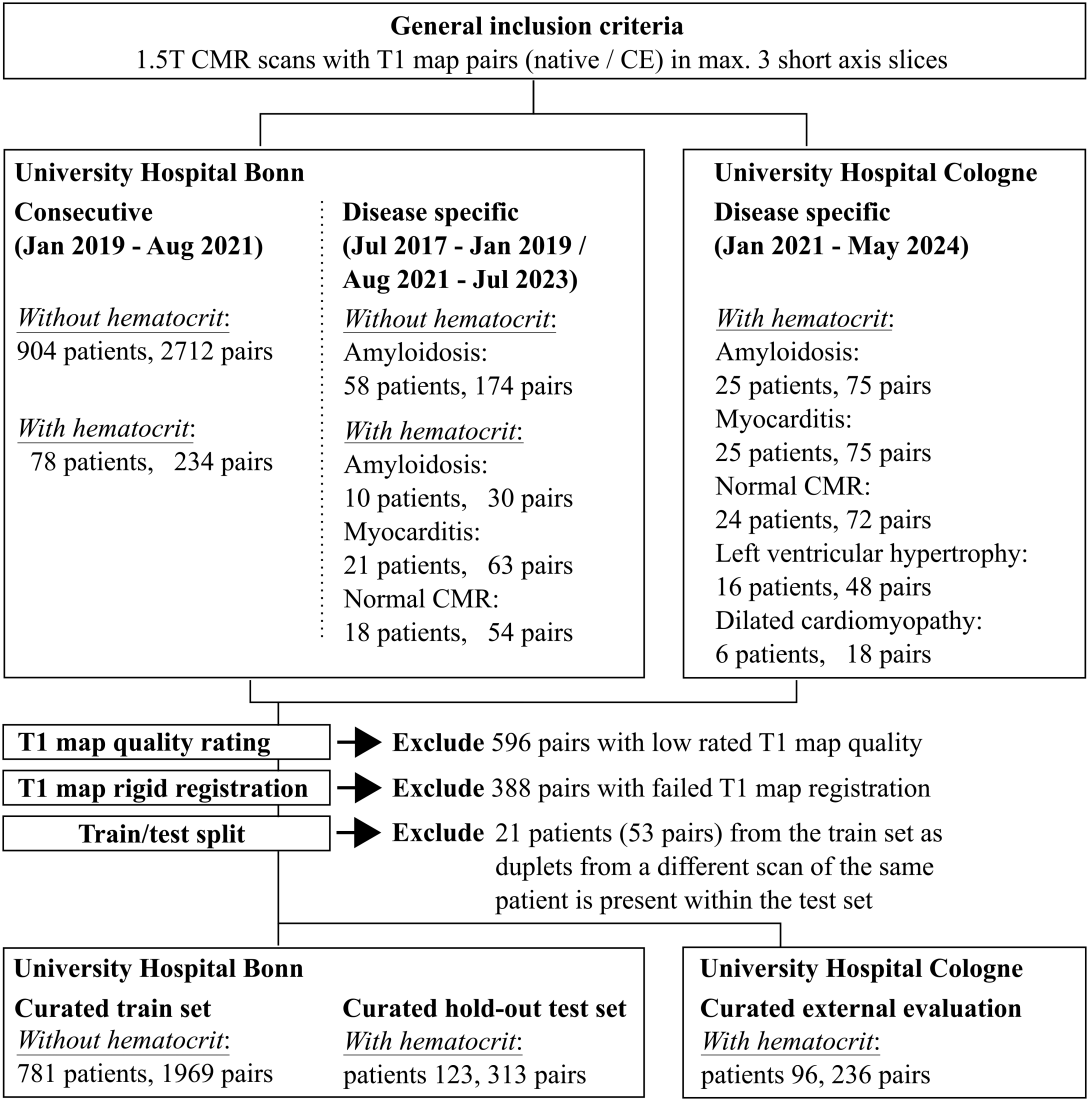
Flowchart illustrating the inclusion and exclusion of native and contrast‐enhanced (CE) T1 map pairs for the training of the generative adversarial network to generate virtual CE T1 maps and for the testing of virtual extracellular volume (ECV) estimations. Only hematocrit values were considered that were derived in a recent blood test within 48 hours before cardiovascular magnetic resonance (CMR).

As the internal data set, 904 patients from the University Hospital Bonn were included (mean age, 49.8±19.6 years; 546 men [60%]; 2282 T1 map pairs). Of those, 123 patients were used as the internal hold‐out test set with recent hematocrit (mean age, 51.9±21.2 years; 81 men; 313 T1 map pairs). Additionally, 96 patients with recent hematocrit (mean age, 51.9±23.1; 65 men; 236 T1 map pairs) from the University Hospital Cologne served as external evaluation. A flowchart for patient selection is given in Figure 2. Patient characteristics for the training and test set are given in Table 1. Examples for T1 maps excluded due to bad T1 map quality or due to infeasible rigid registration due to, for example, mismatch in myocardial contraction can be found in Figures S1 to S4.

**Table 1:** Characteristics of Patients Included in the Hold‐Out Test Set

Basic characteristics	Hold‐out test set	Training set
No. of patients	123	781
Age, y	51.9±21.2	49.5±19.3
Sex, male/female	81/42	465/316
Body mass index, kg/m^2^	25.4±5.0	26.2±6.1
Body surface area, m^2^	1.95±0.25	1.96±0.30
Risk factors, n (%)		
Hypertension	42 (33)	306 (39)
Hypercholesterinemia	40 (31)	229 (29)
Smoking history	33 (26)	200 (25)
Family history of CVD	19 (15)	84 (11)
Diabetes	15 (12)	73 (9)
Obesity	21 (16)	168 (22)
Primary CMR diagnosis, n (%)		
Myocarditis	46 (37)	135 (17)
Normal CMR	25 (20)	328 (42)
Dilated cardiomyopathy	12 (10)	46 (6)
Amyloidosis	11 (9)	68 (9)
Ischemic cardiomyopathy	7 (6)	59 (8)
Pericarditis	6 (5)	40 (5)
Takotsubo/stress edema	5 (4)	2 (<1)
Myocardial fibrosis	4 (3)	33 (4)
Left ventricular hypertrophy	3 (2)	15 (2)
Other	4 (3)	55 (7)
MRI parameters		
LVEDV, mL	177±59	167±64
LVEDV index, mL/m^2^	90±24	85±30
LVESV, mL	89±58	77±55
LVESV index, mL/m^2^	45±25	40±27
LVEF, %	53±14	57±12
Late gadolinium enhancement, n (%)	85 (66)	360 (46)

Diagnoses that occurred in <3 patients of the hold‐out test set were combined under “other.” CMR indicates cardiovascular magnetic resonance; CVD, cardiovascular disease; LVEDV, left ventricular end‐diastolic volume; LVEDV index, left ventricular end‐diastolic volume/body surface area; LVEF, left ventricular ejection fraction; LVESV, left ventricular end‐systolic volume; LVESV index, left ventricular end‐systolic volume/body surface area; and MRI, magnetic resonance imaging.

### GAN for vCE T1 Maps

Mean myocardial CE T1 and vCE T1 relaxation times showed similar mean T1 values (mean CE T1, 372±56 ms versus mean vCE T1, 373±29 ms; *P*=0.37), considering all patients (n=781) from the 5‐fold cross‐validated training set. For patients with ischemic cardiomyopathy (n=59), significant differences were noted (mean CE T1=350±45 ms vs. mean vCE T1=364±29 ms, *P*=0.03). Differences in mean myocardial CE T1 and vCE T1 relaxation times for various CMR findings are shown in Table 2.

**Table 2:** Five‐Fold Cross‐Validation Results for Predicting vCE T1 Maps

CMR diagnosis	CE T1 (ms)	vCE T1 (ms)	*P* value	ΔCE T1 (ms)
All (n=781)	372±56	373±29	0.37	39±33
Normal CMR (n=328)	381±50	382±23	0.57	36±32
Myocarditis (n=135)	371±52	372±22	0.77	40±33
Amyloidosis (n=68)	327±81	330±40	0.69	49±42
Ischemic cardiomyopathy (n=59)	350±45	364±29	0.03^*^	42±29
Dilated cardiomyopathy (n=46)	391±42	389±20	0.71	36±22
Pericarditis (n=40)	378±59	375±26	0.75	40±42
Myocardial fibrosis (n=33)	377±49	377±20	0.96	40±30
Left ventricular hypertrophy (n=15)	371±52	351±15	0.08	43±40
Takotsubo/stress edema (n=2)	352±20	367±6	N/A	16±15
Other (n=55)	373±46	376±20	0.58	32±27

Mean±SD, as well as ΔCE T1 are given for conventional myocardial contrast‐enhanced T1 relaxation times and vCE T1 relaxation times separated for different subgroups on the basis of cardiovascular magnetic resonance diagnosis. *P* values were obtained using a permutation version of the 2‐sided *t* test with 10 000 resamples. Bold numbers indicate statistical significance. vCE indicates virtual contrast‐enhanced.*Statistical significance.

### Evaluation of Contrast‐Free vECV

Considering all patients of the hold‐out test set (n=123), mean myocardial ECV and GAN‐based vECV showed no significant differences (mean ECV: 30.1%±8.0% versus mean vECV, 29.9%±6.4%; *P*=0.49). In patients with amyloidosis (n=11), significant differences were noted (mean ECV, 48.2%±11.6% versus mean vECV, 42.2±9.5%; *P*<0.01). Compared with GAN‐based vECV, RFR‐based vECV showed higher differences in amyloidosis (mean ECV, 48.2%±11.6% versus mean vECV, 34.9%±6.3%; *P*<0.01) and also in normal CMR (mean ECV, 25.5%±3.2% versus mean vECV, 26.9%±3.1%; *P*=0.01). Differences in mean myocardial ECV and vECV for various CMR findings and both machine‐learning methods are given in Table 3.

**Table 3:** Hold‐Out Test Set Results for ECV and vECV Fraction

CMR diagnosis	ECV (%)	vECV (%)	*P* value	ΔECV (%)
Generative adversarial network				
All patients (n=123)	30.1±8.0	29.9±6.4	0.49	2.4±2.8
Myocarditis (n=46)	29.5±4.8	29.4±4.1	0.83	2.0±1.5
Normal CMR (n=25)	25.4±3.1	26.1±2.7	0.16	1.9±1.4
Dilated cardiomyopathy (n=12)	30.8±4.8	31.8±3.7	0.26	2.4±1.8
Amyloidosis (n=11)	48.2±11.6	42.2±9.5	<0.01^*^	6.2±6.0
Ischemic cardiomyopathy (n=7)	25.6±3.2	25.3±4.9	0.81	1.9±0.7
Pericarditis (n=6)	28.6±2.0	28.0±2.4	0.62	2.1±1.6
Takotsubo/stress edema (n=5)	31.7±5.1	32.0±4.7	0.75	1.1±0.8
Myocardial fibrosis (n=4)	27.5±2.3	25.8±1.4	N/A	2.6±0.7
Left ventricular hypertrophy (n=3)	28.9±1.2	36.3±3.6	N/A	7.4±4.5
Other (n=4)	25.6±2.3	25.6±2.4	N/A	0.1±0.1
Random forest regressor				
All patients (n=123)	30.1±8.0	29.7±5.4	0.50	3.7±5.2
Myocarditis (n=46)	29.5±4.8	30.3±5.6	0.13	2.5±2.2
Normal CMR (n=25)	25.5±3.2	26.9±3.1	0.01^*^	2.4±1.9
Dilated cardiomyopathy (n=12)	30.8±4.8	31.0±2.4	0.93	3.2±3.2
Amyloidosis (n=11)	48.1±11.8	34.9±6.3	<0.01^*^	14.7±10.8
Ischemic cardiomyopathy (n=7)	25.6±3.2	25.5±4.0	0.94	1.7±0.9
Pericarditis (n=6)	28.6±2.0	29.5±3.4	0.62	2.9±1.9
Takotsubo/stress edema (n=5)	31.7±5.1	34.6±6.2	0.12	3.3±2.0
Myocardial fibrosis (n=4)	27.5±2.3	26.8±3.4	N/A	1.7±1.0
Left ventricular hypertrophy (n=3)	28.9±1.2	31.2±5.6	N/A	5.5±3.7
Other (n=4)	25.6±2.3	25.6±3.4	N/A	1.2±0.9

ECV and vECV were calculated on the basis of contrast‐enhanced T1 map and virtual contrast‐enhanced T1 values generated by a generative adversarial network and by a random forest regressor. Mean±SD, as well as mean of the difference per patient are given for ECV and vECV separated into subgroups on the basis of CMR diagnosis. *P* values were obtained using a permutation version of the 2‐sided *t* test with 10 000 resamples.  CMR indicates cardiovascular magnetic resonance; ECV, extracellular volume; ΔECV, mean of the difference in extracellular volume per patient; and vECV, virtual extracellular volume.

*Statistical significance.

We calculated GAN‐based vECV values for 81 native T1 map pairs, which were excluded from comparison with conventional ECV because registration with the conventional CE T1 map failed due to dissimilar slice or myocardial contraction (normal CMR n=42, mean vECV, 27.7%±4.5%; myocarditis n=81, mean vECV, 29.9%±4.9%; amyloidosis n=11, mean vECV, 42.2%±8.4%; dilated cardiomyopathy n=1, vECV=39.3%).

Similar diagnostic performance of conventional ECV and vECV for the detection of patients with myocarditis versus patients with normal CMR was observed (hold‐out test: ECV AUC, 0.77 [95% CI, 0.65–0.87] versus vECV AUC, 0.76 [95% CI, 0.63–0.86]; *P*=0.76). Diagnostic performance results of vECV for the detection of cardiac amyloidosis are given in Table 4.

**Table 4:** Diagnostic Performance of Myocardial ECV and vECV Fraction

Variable	Sensitivity (%)	Specificity (%)	Accuracy (%)	McNemar *P* value	AUC	Delong *P* value
Myocarditis						
ECV	54 (42–69)	92 (80–100)	68 (56–78)		0.77 (0.65–0.87)	
vECV	52 (37–67)	80 (64–96)	62 (50–73)		0.76 (0.63–0.86)	
Difference	2 (0–16)	12 (0–31)	6 (0–15)	1.00	0.01 (0–0.10)	0.76
Amyloidosis						
ECV	100 (100–100)	92 (80–100)	94 (86–100)		0.99 (0.97–1)	
vECV	91 (70–100)	80 (62–95)	83 (69–94)		0.96 (0.86–1)	
Difference	9 (0–30)	12 (0–28)	11 (0–25)	1.00	0.03 (0–0.12)	0.52

Diagnostic performance was evaluated for patients with myocarditis or amyloidosis in the hold‐out test set. Sensitivity, specificity, AUC, as well as their absolute mean differences are given. Data in parentheses are 95% CIs. Differences in ECV and vECV were determined by a McNemar's test and differences in AUC were determined by Delong's test. Both tests were conducted as permutation tests with 10 000 resamples. AUC indicates area under the receiver operating curve; ECV, extracellular volume; and vECV, virtual extracellular volume.

**Figure 3:**
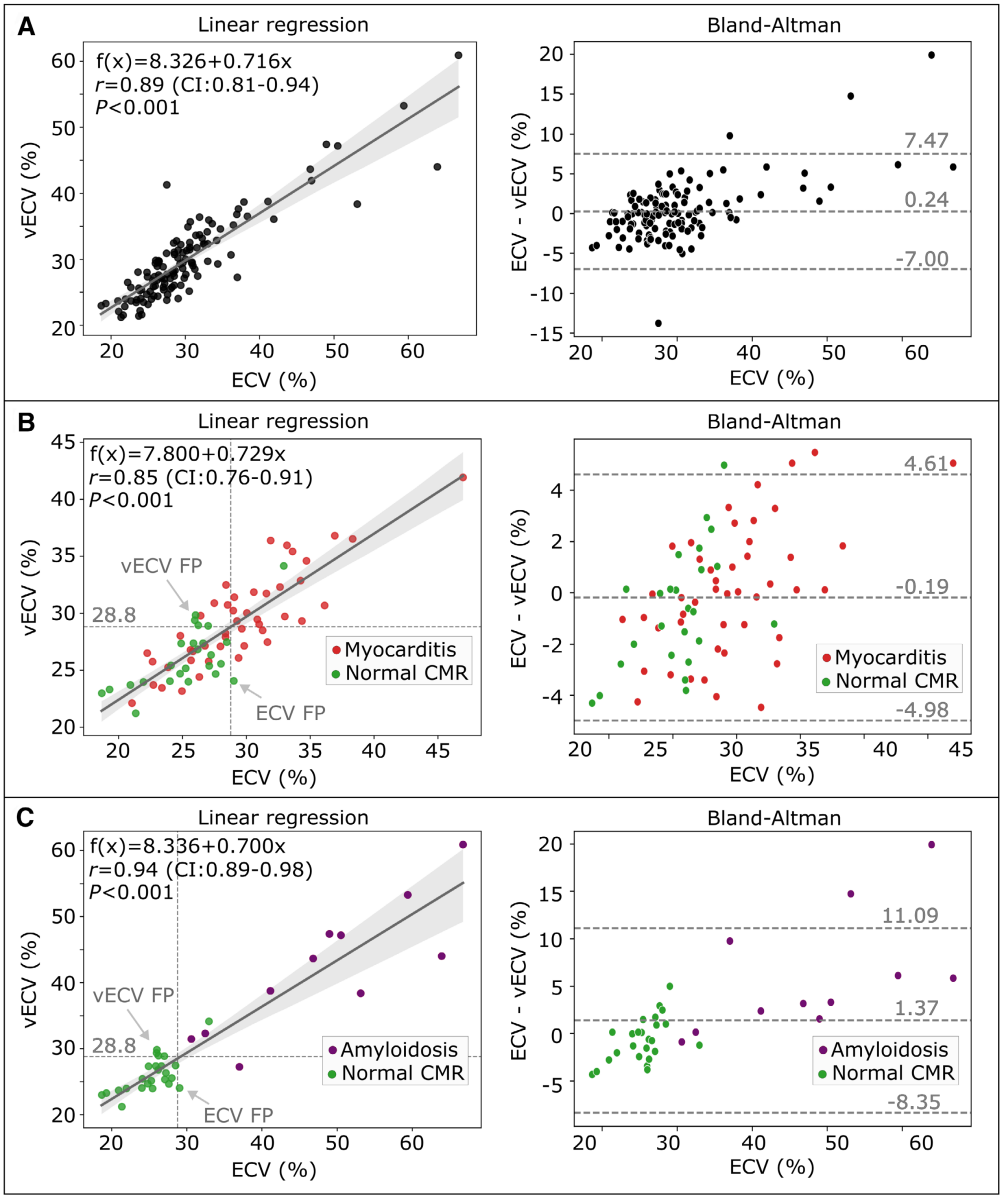
Linear regression and Bland–Altman analysis between conventional extracellular volume (ECV) and virtual extracellular volume (vECV) fraction for (A) using all test cases, (B) only using patients with normal cardiovascular magnetic resonance (CMR) results and patients with myocarditis and (C) only using patients with normal CMR and patients with amyloidosis. In the linear regression plot, the applied ECV threshold of 28.8% for differentiating normal CMR to diseased is indicated by dotted lines. Cases that are detected false positive (FP) by vECV but true negative by ECV and vice versa are indicated by arrows. Conventional ECV was used as estimator of true ECV (*x* axis).

Correlation between conventional ECV and vECV was high in the hold‐out test (all patients: *r*=0.89 [95% CI, 0.81–0.94], *P* < 0.001; myocarditis subcohort: *r*=0.85 [95% CI, 0.76–0.91], *P*<0.001; amyloidosis subcohort: *r*=0.94 [95% CI, 0.89–0.98], *P*<0.001) (see Figure 3).

**Figure 4:**
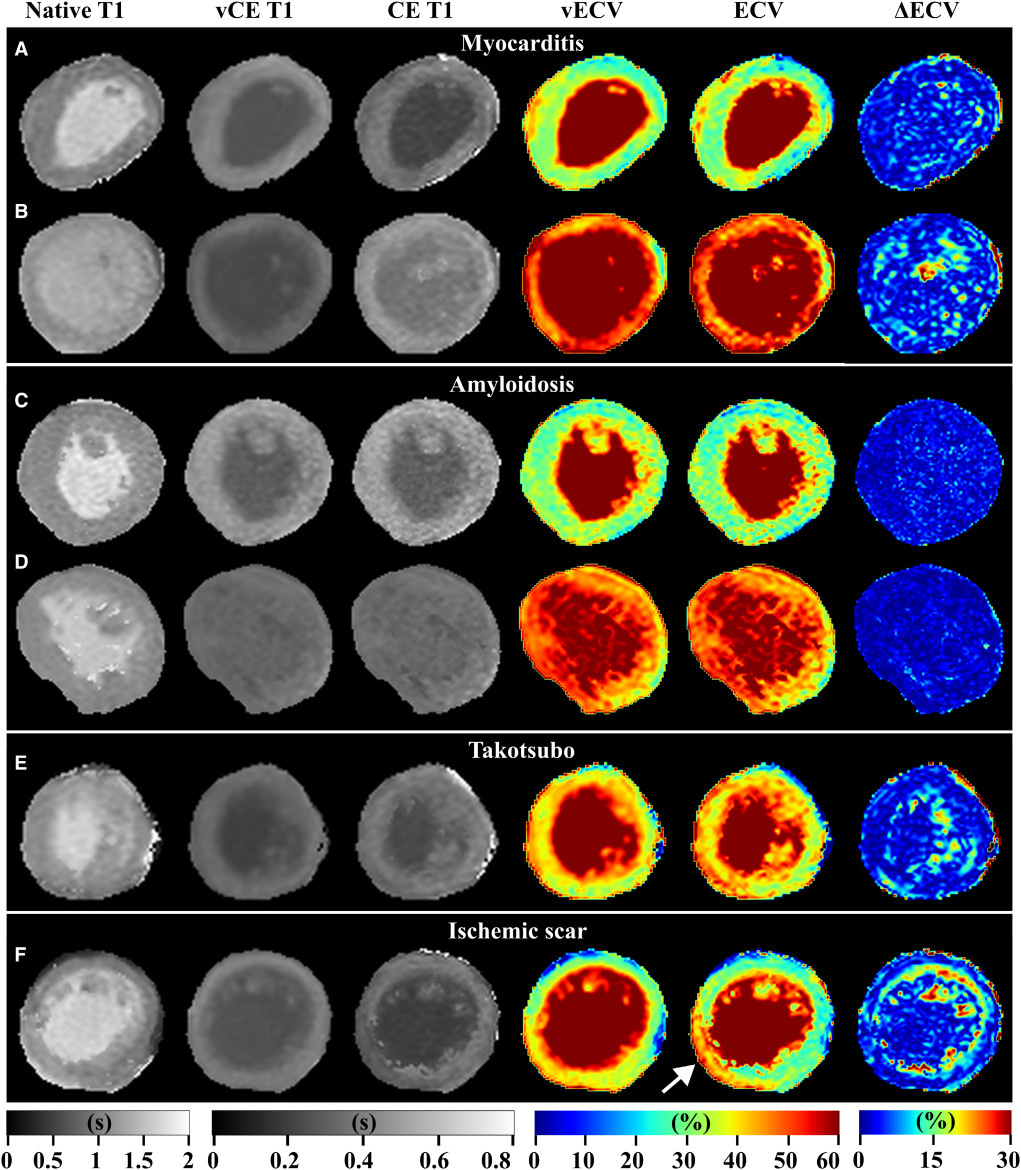
Clinical examples from the hold‐out test set with global and focal agreement between conventional extracellular volume (ECV) and virtual extracellular volume (vECV). Examples for patients with diagnosis of myocarditis (**A** and **B**), amyloidosis (**C** and **D**), Takotsubo syndrome (**E**) and ischemic scar (**F**, white arrow) are shown. Native, contrast‐enhanced (CE) and virtual contrast‐enhanced (vCE) T1 maps, as well as ECV, vECV, and pixel‐wise ΔECV maps are shown.

**Figure 5:**
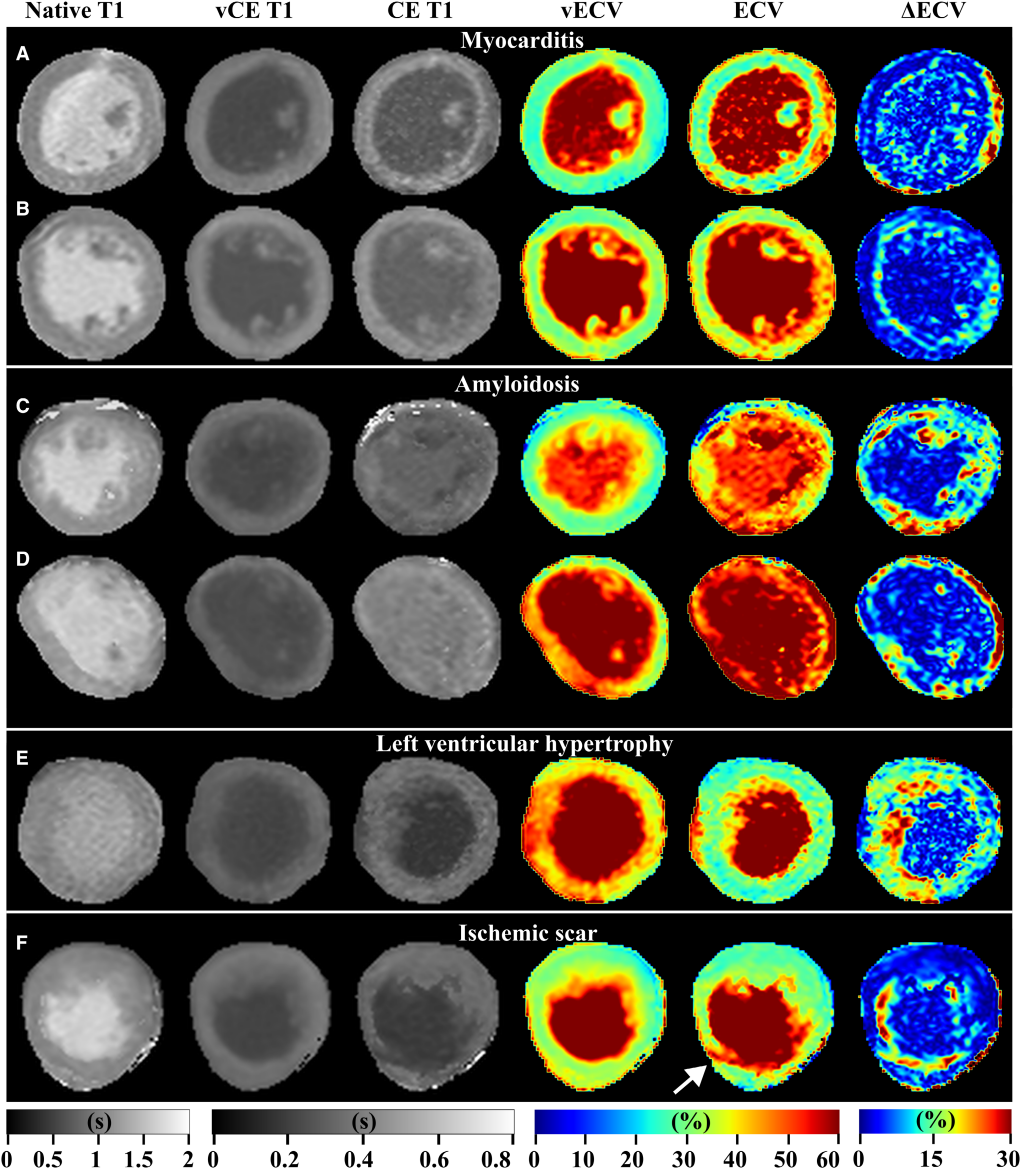
Clinical examples from the hold‐out test set with low global and focal agreement between conventional extracellular volume (ECV) and virtual extracellular volume (vECV) maps. Examples for patients with diagnosis of myocarditis (**A** and **B**), amyloidosis (**C** and **D**), Takotsubo syndrome (**E**) and ischemic scar (**F**, white arrow) are shown. Native, contrast‐enhanced (CE) and virtual contrast‐enhanced (vCE) T1 maps, as well as ECV, vECV, and pixel‐wise ΔECV maps are shown.

Examples of native, CE, and vCE T1 maps, as well as ECV and vECV maps are given in Figures 4 and 5. Seventy‐three of 123 (59%) patients in the hold‐out test set had focal or diffuse lesions on the conventional ECV map. Of those, 38 of 73 (52%) patients featured also a lesion in the same location in the vECV. In 8 of 3 (11%) patients the vECV map showed also localized lesions, but at a different location than the lesion in the conventional ECV map. In 3 of 50 (4%) patients who had no lesion in the conventional ECV map, the vECV map falsely featured a focal lesion. Detailed results of visual evaluation of focal and diffuse lesions on the ECV maps separated for diagnosis are given in Table 5.

**Table 5:** Results of Visual Assessment of Focal or Diffuse Lesions

Diagnosis	ECV with lesions, n (%)	Rating A, n (%)	Rating B, n (%)	Rating C, n (%)	ECV without lesion, n (%)	Rating D, n (%)
All patients (n=123)	73 (59)	38 (52)	8 (11)	34 (47)	50 (41)	3 (4)
Myocarditis (n=46)	34 (74)	13 (38)	4 (12)	20 (59)	12 (26)	1 (3)
Dilated cardiomyopathy (n=12)	9 (75)	6 (67)	1 (11)	3 (33)	3 (25)	2 (22)
Amyloidosis (n=11)	11 (100)	10 (91)	0 (0)	1 (9)	0 (0)	…
Ischemic cardiomyopathy (n=7)	5 (71)	2 (40)	0 (0)	3 (60)	2 (29)	0 (0)
Pericarditis (n=6)	3 (50)	1 (33)	0 (0)	2 (67)	3 (50)	0 (0)
Takotsubo/stress edema (n=5)	4 (80)	3 (75)	1 (25)	1 (25)	1 (20)	0 (0)
Myocardial fibrosis (n=4)	4 (100)	0 (0)	0 (0)	4 (100)	0 (0)	…
Left ventricular hypertrophy (n=3)	3 (100)	3 (100)	2 (67)	0 (0)	0 (0)	…
Other (n=4)	0 (0)	0 (0)	0 (0)	0 (0)	0 (0)	…

Focal or diffuse lesions present in the conventional ECV and vECV maps of the patients were assessed in the hold‐out test set. For each patient, the lesions that are present in the vECV maps were rated into the following categories: (A) lesions present in vECV in same location as in conventional ECV map; (B) lesions present in vECV in different location than in conventional ECV map; (C) no lesions present in vECV but in conventional ECV; and (D) lesions hallucinated, as none are present in conventional ECV map. Note that a patient can feature multiple lesions and thereby occur in the A and B rating simultaneously. For lesions in vECV, the proportion to the number of lesions in the conventional ECV is also given in brackets. ECV indicates extracellular volume; and vECV, virtual extracellular volume.

### External Evaluation of Contrast‐Free vECV

**Figure 6:**
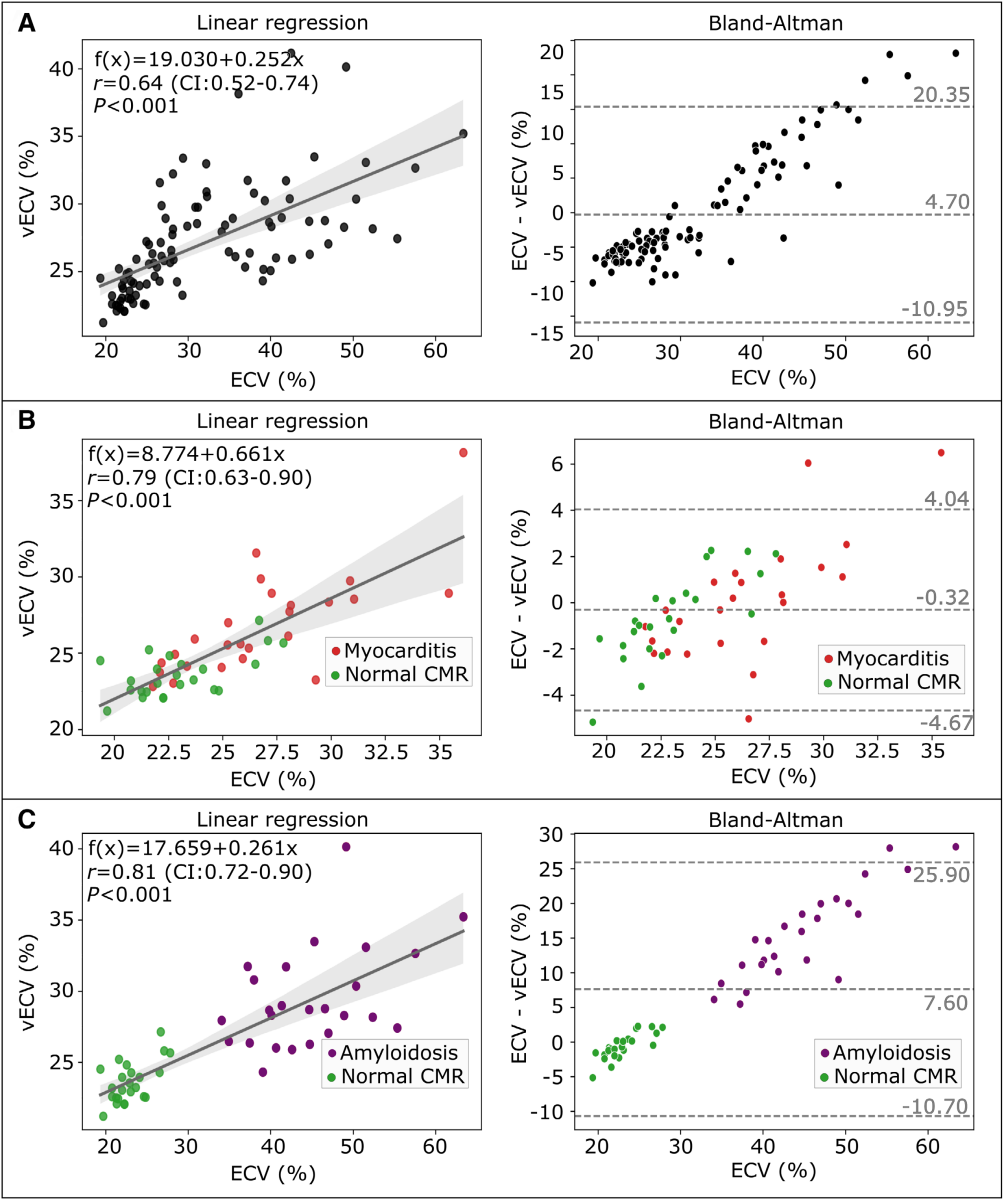
Linear regression and Bland–Altman analysis between conventional extracellular volume (ECV) and virtual extracellular volume (vECV) fraction for (A) using all cases of the external validation cohort, (B) only using patients with normal cardiovascular magnetic resonance (CMR) results and patients with myocarditis and (C) only using patients with normal CMR and patients with amyloidosis. Conventional ECV was used as estimator of true ECV (*x* axis).

Differences in mean myocardial ECV and vECV are shown in Table 6 and linear regression and Bland‐Altman analysis are shown in Figure 6. vECV based on the GAN developed on internal T1 maps generalized well to external T1 maps in patients with normal CMR (mean ECV, 23.0%±2.2% versus mean vECV, 23.6%±1.4%; *P*=0.12), with myocarditis (mean ECV, 26.8%±3.7% versus mean vECV, 26.8±3.3%; *P*=0.94) and with dilated cardiomyopathy (mean ECV, 35.3%±4.2% versus mean vECV, 32.2%±4.2%; *P*=0.06). However, as in the internal hold‐out test set, ECV and vECV differed for amyloidosis (mean ECV, 45.0%±7.2% versus mean vECV, 29.5%±3.4%; *P* < 0.01).

**Table 6:** External Evaluation of vECV Compared With ECV Fraction

CMR diagnosis	ECV (%)	vECV (%)	*P* value	ΔECV (%)
All patients (n=96)	31.7±9.9	27.0±3.9	<0.01^*^	6.0±7.1
Myocarditis (n=25)	26.8±3.7	26.8±3.3	0.94	1.9±1.7
Amyloidosis (n=25)	45.0±7.2	29.5±3.4	<0.01^*^	15.5±6.4
Normal (n=24)	23.0±2.2	23.6±1.4	0.12	1.5±1.2
LVH (n=16)	30.7±6.1	26.8±3.1	0.03^*^	5.3±5.0
Dilated cardiomyopathy (n=6)	35.3±4.2	32.2±4.2	0.06	3.3±3.1

ECV and vECV were calculated on the basis of contrast‐enhanced T1 map and virtual contrast‐enhanced T1 values generated by the generative adversarial network. Mean±SD, as well as ΔECV are given for ECV and vECV separated into subgroups on the basis of cardiovascular magnetic resonance diagnosis. *P* values were obtained using a permutation version of the 2‐sided *t* test with 10 000 resamples. ECV indicates extracellular volume; ΔECV, mean of the difference in extracellular volume per patient; and vECV, virtual extracellular volume.

*Statistical significance.

## Discussion

In this explorative proof‐of‐principle study, we investigated the potential and limitations of deep learning for generating virtual CE T1 maps from native T1 maps to enable GBCA‐free estimation of myocardial ECV. We could show that mean myocardial vECV values generated by a GAN had a substantial agreement with conventional ECV (hold‐out test: *r*=0.89 [95% CI, 0.81–0.94]; ΔECV, 2.4%±2.8%) and that the application of vECV had similar diagnostic performance for the diagnosis of myocarditis and amyloidosis compared with conventional ECV (hold‐out test: myocarditis ECV AUC, 0.77 [95% CI, 0.65–0.87] versus vECV AUC, 0.76 [95% CI, 0.63–0.86]; *P*=0.76; amyloidosis ECV AUC, 0.99 [95% CI, 0.97–1] versus vECV AUC, 0.96 [95% CI,0.86–1.00]; *P*=0.52). We could show that the GAN‐based vECV had lower deviations to ECV compared with the application of random forest algorithms, indicating that the GAN‐based T1 map generation does not simply represent a mapping of native to CE T1 values. Furthermore, we observed that the GAN model solely trained on internal T1 maps generalized well on an external data set in patients with normal CMR, myocarditis, and dilated cardiomyopathy. However, lower vECV values were observed in patients with amyloidosis in the internal hold‐out test and in the external evaluation. One reason for this may be the relatively low proportion of patients with amyloidosis (8.7%) in the consecutively derived training data, which is lower than in patients with myocarditis (17.3%) or normal CMR (42.0%), for example. Nevertheless, the promising results of this proof‐of‐principle study for diseases with good representation motivate further exploring of deep learning for contrast‐free vECV estimation in further studies with larger training data sets acquired from multiple centers, specifically targeting diseases with high ECV variance. Nowadays, myocardial mapping plays an important role in the diagnosis of patients with suspected cardiomyopathies as it increases diagnostic accuracy and reading confidence of CMR.^34^ However, this might be especially true for native myocardial T1 and T2 mapping, as the clinical diagnostic value for an additional ECV assessment might be less impactful,^4^, ^34^ possibly due to a more cumbersome application of ECV in a “real world” setting. Even though ECV is listed as an independent T1 criterion in the 2018 Lake Louise criteria for the diagnosis of acute myocarditis, the diagnostic yield of ECV in acute myocarditis is lower than native T1 and T2 relaxation times.^4^, ^35^ So far, ECV has not clearly demonstrated an incremental diagnostic value beyond late gadolinium‐enhanced and native mapping techniques in myocarditis CMR.^35^ In the absence of significant myocardial inflammation, however, ECV is correlated with diffuse myocardial fibrosis.^36^ This has prognostic implications in patients with left ventricular hypertrophy (eg, due to severe aortic stenosis).^37^ In this regard, ECV is also important for the evaluation and detection of infiltrative and storage myocardial diseases including cardiac amyloidosis.^9^ However, the assessment of ECV has some drawbacks and barriers in clinical routine, as additional CE T1 maps must be acquired (CE T1 maps are often acquired many minutes after the native maps, which limits registration in clinical routine), and timely hematocrit assessment is needed for accurate estimation.^38^ Therefore, many centers might not routinely acquire CE T1 maps in clinical protocols and perform hematocrit blood test values before imaging due to additional costs.^38^ In this regard, deep learning methods for vECV estimation, as proposed in our study, might facilitate a broader use of ECV in the future, as such algorithms can provide vECV maps immediately at the point of reading, like a point‐of‐care test, when the information is really needed. We demonstrated this by generating vECV for native T1 maps for which conventional ECV could not be derived due to infeasible registration with the CE T1 map. Together with other algorithms, which can calculate virtual late gadolinium enhanced images,^16^, ^17^ faster and less expensive CMR without the use of GBCAs might become accessible in the future.

The not widespread acquisition of CE T1 maps in combination with missing hematocrit blood test values leads to a limited number of ground truth ECV in clinical databases compared with established imaging sequences such as late gadolinium enhanced. To avoid this limitation, we propose GBCA‐free estimation of vECV by generating vCE T1 maps from native T1 maps. However, besides still requiring recent hematocrit blood values, this approach has some disadvantages: Imaging‐specific factors, such as the time between contrast agent administration and CE T1 map acquisition, but also patient‐dependent factors that influence how rapid the contrast agent is distributed and excreted, such as renal function, are subject to a certain degree of variability and influence CE myocardial T1.^3^ To compensate for such factors, the CE T1 map in our study was acquired after a fixed interval of 10 minutes after contrast administration. In driven equilibrium ECV determination, the variability stemming from contrast agent distribution is normalized by bringing differences of native and CE R1 values of the myocardium and the blood pool into relation, which means that a deep learning model does not need additional information about possible influencing factors. This motivates future multicenter studies including more patients with appropriate CMR protocols and hematocrit to investigate a direct generation of vECV.

The proposed method has further limitations. On visual assessment of focal or diffuse lesions, we found out that only half of the lesions that were identified on the conventional ECV map were also present in the vECV map. Contrast agents provide additional information about the ECV and are especially important for identification of small lesions. Thus, the calculation of a perfect contrast‐free vECV representing all small focal lesions is likely not possible solely on the basis of native T1 maps. Also, the myocardium of the vCE T1 and vECV maps have a “smoother” appearance, which is common for images generated by pixel‐to‐pixel GANs that optimize pixel‐wise losses such as mean absolute distance or Euclidean distance.^39^ In addition, in 8 patients with lesions in the conventional ECV, lesions were found in the vECV at other positions in the myocardium, and in 3 patients a lesion was falsely present in the vECV map as no lesions were found in the conventional ECV. The risk of hallucination of image content of GANs, which is facilitated by training with adversarial objectives by deceiving the discriminator, is particularly critical for applications in the medical field. One way to suppress the occurrence of hallucinated image content is to combine pixel‐wise and adversarial losses, as applied in the current work.^40^ Also, we did not investigate recently presented diffusion and vision transformer‐based image‐generation approaches.^41^ However, vision transformers were shown to be only superior to convolutional neural networks, if vast amounts of training data were available, which was not the case in the current study.^42^ Also, the standard U‐Net already demonstrated capabilities for generation of contrast‐agent CMR images in previous studies.^16^, ^17^ Therefore, we decided to evaluate U‐Net–based GANs in this proof‐of‐principle study. Future work could compare transformer‐based diffusion models with respect to quality and reliability of local tissue features. Finally, although the internal hold‐out test and the external validation had different modified Look‐Locker inversion‐recovery acquisition schemes, the validation was performed with a single vendor and exclusively with 1.5T (1.5T Ingenia, Philips Healthcare).

In conclusion, deep learning–based contrast agent–free myocardial ECV estimation by generating vCE T1 maps is feasible and offers a clinically applicable approach for the determination of mean myocardial ECV values. This technique might ultimately facilitate faster CMR and increase patient safety, especially for those with impaired renal function. Further studies are warranted to ultimately determine the clinical implications of deep learning–based image generation in CMR.

## Sources of Funding

This work was supported by the Open Access Publication Fund of the University of Bonn.

## Disclosures

None.

## Supporting information

Data S1–S4
Figures S1–S4

## References

[1] Arbelo E , Protonotarios A , Gimeno JR , Arbustini E , Barriales‐Villa R , Basso C , Bezzina CR , Biagini E , Blom NA , de Boer RA , et al. 2023 ESC guidelines for the management of cardiomyopathies: developed by the task force on the management of cardiomyopathies of the European Society of Cardiology (ESC). Eur Heart J. 2023;44:3503–3626. doi: 10.1093/eurheartj/ehad194 37622657

[2] Luetkens JA , Homsi R , Dabir D , Kuetting DL , Marx C , Doerner J , Schlesinger‐Irsch U , Andrié R , Sprinkart AM , Schmeel FC , et al. Comprehensive cardiac magnetic resonance for short‐term follow‐up in acute myocarditis. J Am Heart Assoc. 2016;5:e003603. doi: 10.1161/JAHA.116.003603 27436306 PMC5015395

[3] Haaf P , Garg P , Messroghli DR , Broadbent DA , Greenwood JP , Plein S . Cardiac T1 mapping and extracellular volume (ECV) in clinical practice: a comprehensive review. J Cardiovasc Magn Reson. 2016;18:89. doi: 10.1186/s12968-016-0308-4 27899132 PMC5129251

[4] Luetkens JA , Faron A , Isaak A , Dabir D , Kuetting D , Feisst A , Schmeel FC , Sprinkart AM , Thomas D . Comparison of original and 2018 Lake Louise criteria for diagnosis of acute myocarditis: results of a validation cohort. Radiol: Cardiothorac Imaging. 2019;1:e190010. doi: 10.1148/ryct.2019190010 33778510 PMC7978026

[5] Luetkens JA , Homsi R , Sprinkart AM , Doerner J , Dabir D , Kuetting DL , Block W , Andrié R , Stehning C , Fimmers R , et al. Incremental value of quantitative CMR including parametric mapping for the diagnosis of acute myocarditis. Eur Heart J Cardiovasc Imaging. 2016;17:154–161. doi: 10.1093/ehjci/jev246 26476398 PMC4882886

[6] Cui Y , Cao Y , Song J , Dong N , Kong X , Wang J , Yuan Y , Zhu X , Yan X , Greiser A , et al. Association between myocardial extracellular volume and strain analysis through cardiovascular magnetic resonance with histological myocardial fibrosis in patients awaiting heart transplantation. J Cardiovasc Magn Reson. 2018;20:25. doi: 10.1186/s12968-018-0445-z 29681243 PMC5911945

[7] Luetkens JA , Klein S , Träber F , Schmeel FC , Sprinkart AM , Kuetting DLR , Block W , Uschner FE , Schierwagen R , Hittatiya K , et al. Quantification of liver fibrosis at T1 and T2 mapping with extracellular volume fraction MRI: preclinical results. Radiology. 2018;288:748–754. doi: 10.1148/radiol.2018180051 29944086

[8] Isaak A , Praktiknjo M , Jansen C , Faron A , Sprinkart AM , Pieper CC , Chang J , Fimmers R , Meyer C , Dabir D , et al. Myocardial fibrosis and inflammation in liver cirrhosis: MRI study of the liver‐heart axis. Radiology. 2020;297:51–61. doi: 10.1148/radiol.2020201057 32808886

[9] Martinez‐Naharro A , Patel R , Kotecha T , Karia N , Ioannou A , Petrie A , Chacko LA , Razvi Y , Ravichandran S , Brown J , et al. Cardiovascular magnetic resonance in light‐chain amyloidosis to guide treatment. Eur Heart J. 2022;43:4722–4735. doi: 10.1093/eurheartj/ehac363 36239754 PMC9712028

[10] Grobner T . Gadolinium—a specific trigger for the development of nephrogenic fibrosing dermopathy and nephrogenic systemic fibrosis? Nephrol Dial Transplant. 2006;21:1104–1108. doi: 10.1093/ndt/gfk062 16431890

[11] Kanda T , Fukusato T , Matsuda M , Toyoda K , Oba H , Kotoku J , Haruyama T , Kitajima K , Furui S . Gadolinium‐based contrast agent accumulates in the brain even in subjects without severe renal dysfunction: evaluation of autopsy brain specimens with inductively coupled plasma mass spectroscopy. Radiology. 2015;276:228–232. doi: 10.1148/radiol.2015142690 25942417

[12] Do C , DeAguero J , Brearley A , Trejo X , Howard T , Escobar GP , Wagner B . Gadolinium‐based contrast agent use, their safety, and practice evolution. Kidney360. 2020;1:561–568. doi: 10.34067/KID.0000272019 34423308 PMC8378745

[13] Mallio CA , Radbruch A , Deike‐Hofmann K , van der Molen AJ , Dekkers IA , Zaharchuk G , Parizel PM , Beomonte Zobel B , Quattrocchi CC . Artificial intelligence to reduce or eliminate the need for gadolinium‐based contrast agents in brain and cardiac MRI: a literature review. Investig Radiol. 2023;58:746–753. doi: 10.1097/RLI.0000000000000983 37126454

[14] Haase R , Pinetz T , Kobler E , Paech D , Effland A , Radbruch A , Deike‐Hofmann K . Artificial contrast: deep learning for reducing gadolinium‐based contrast agents in neuroradiology. Investig Radiol. 2023;58:539–547. doi: 10.1097/RLI.0000000000000963 36822654

[15] Haase R , Pinetz T , Bendella Z , Kobler E , Paech D , Block W , Effland A , Radbruch A , Deike‐Hofmann K . Reduction of gadolinium‐based contrast agents in MRI using convolutional neural networks and different input protocols: limited interchangeability of synthesized sequences with original full‐dose images despite excellent quantitative performance. Investig Radiol. 2023;58:420. doi: 10.1097/RLI.0000000000000955 36735399

[16] Zhang Q , Burrage MK , Lukaschuk E , Shanmuganathan M , Popescu IA , Nikolaidou C , Mills R , Werys K , Hann E , Barutcu A , et al. Toward replacing late gadolinium enhancement with artificial intelligence virtual native enhancement for gadolinium‐free cardiovascular magnetic resonance tissue characterization in hypertrophic cardiomyopathy. Circulation. 2021;144:589–599. doi: 10.1161/CIRCULATIONAHA.121.054432 34229451 PMC8378544

[17] Zhang Q , Burrage MK , Shanmuganathan M , Gonzales RA , Lukaschuk E , Thomas KE , Mills R , Leal Pelado J , Nikolaidou C , Popescu IA , et al. Artificial intelligence for contrast‐free MRI: scar assessment in myocardial infarction using deep learning–based virtual native enhancement. Circulation. 2022;146:1492–1503. doi: 10.1161/CIRCULATIONAHA.122.060137 36124774 PMC9662825

[18] Messroghli DR , Radjenovic A , Kozerke S , Higgins DM , Sivananthan MU , Ridgway JP . Modified look‐locker inversion recovery (MOLLI) for high‐resolution T1 mapping of the heart. Magn Reson Med. 2004;52:141–146. doi: 10.1002/mrm.20110 15236377

[19] Caforio ALP , Pankuweit S , Arbustini E , Basso C , Gimeno‐Blanes J , Felix SB , Fu M , Heliö T , Heymans S , Jahns R , et al. Current state of knowledge on aetiology, diagnosis, management, and therapy of myocarditis: a position statement of the European Society of Cardiology Working Group on myocardial and pericardial diseases. Eur Heart J. 2013;34:2636–2648. doi: 10.1093/eurheartj/eht210 23824828

[20] Garcia‐Pavia P , Rapezzi C , Adler Y , Arad M , Basso C , Brucato A , Burazor I , Caforio ALP , Damy T , Eriksson U , et al. Diagnosis and treatment of cardiac amyloidosis: a position statement of the ESC working group on myocardial and pericardial diseases. Eur Heart J. 2021;42:1554–1568. doi: 10.1093/eurheartj/ehab072 33825853 PMC8060056

[21] Ronneberger O , Fischer P , Brox T . U‐net: convolutional networks for biomedical image segmentation. Medical Image Computing and Computer‐Assisted Intervention–MICCAI. In: Navab N , Hornegger J , Wells WM , Frangi A , eds. Springer International Publishing;2015:234–241. doi: 10.1007/978-3-319-24574-4_28

[22] Nowak S , Mesropyan N , Faron A , Block W , Reuter M , Attenberger UI , Luetkens JA , Sprinkart AM . Detection of liver cirrhosis in standard T2‐weighted MRI using deep transfer learning. Eur Radiol. 2021;31:8807–8815. doi: 10.1007/s00330-021-07858-1 33974149 PMC8523404

[23] Nowak S , Theis M , Wichtmann BD , Faron A , Froelich MF , Tollens F , Geißler HL , Block W , Luetkens JA , Attenberger UI , et al. End‐to‐end automated body composition analyses with integrated quality control for opportunistic assessment of sarcopenia in CT. Eur Radiol. 2022;32:3142–3151. doi: 10.1007/s00330-021-08313-x 34595539 PMC9038788

[24] Theis M , Tonguc T , Savchenko O , Nowak S , Block W , Recker F , Essler M , Mustea A , Attenberger U , Marinova M , et al. Deep learning enables automated MRI‐based estimation of uterine volume also in patients with uterine fibroids undergoing high‐intensity focused ultrasound therapy. Insights Imaging. 2023;14:1. doi: 10.1186/s13244-022-01342-0 36600120 PMC9813298

[25] Nowak S , Henkel A , Theis M , Luetkens J , Geiger S , Sprinkart AM , Pieper CC , Attenberger UI . Deep learning for standardized, MRI‐based quantification of subcutaneous and subfascial tissue volume for patients with lipedema and lymphedema. Eur Radiol. 2023;33:884–892. doi: 10.1007/s00330-022-09047-0 35976393 PMC9889496

[26] Pasumarthi S , Tamir JI , Christensen S , Zaharchuk G , Zhang T , Gong E . A generic deep learning model for reduced gadolinium dose in contrast‐enhanced brain MRI. Magn Reson Med. 2021;86:1687–1700. doi: 10.1002/mrm.28808 33914965

[27] Wang Z , Bovik AC , Sheikh HR , Simoncelli EP . Image quality assessment: from error visibility to structural similarity. IEEE Trans Image Process. 2004;13:600–612. doi: 10.1109/TIP.2003.819861 15376593

[28] Flett AS , Hayward MP , Ashworth MT , Hansen MS , Taylor AM , Elliott PM , McGregor C , Moon JC . Equilibrium contrast cardiovascular magnetic resonance for the measurement of diffuse myocardial fibrosis. Circulation. 2010;122:138–144. doi: 10.1161/CIRCULATIONAHA.109.930636 20585010

[29] Virtanen P , Gommers R , Oliphant TE , Haberland M , Reddy T , Cournapeau D , Burovski E , Peterson P , Weckesser W , Bright J , et al. SciPy 1.0: fundamental algorithms for scientific computing in python. Nat Methods. 2020;17:261–272. doi: 10.1038/s41592-019-0686-2 32015543 PMC7056644

[30] Pedregosa F , Varoquaux G , Gramfort A , Michel V , Thirion B , Grisel O , Blondel M , Prettenhofer P , Weiss R , Dubourg V , et al. Scikit‐learn: machine learning in python. J Mach Learn Res. 2011;12:2825–2830.

[31] Seabold S , Perktold J . Statsmodels: econometric and statistical modeling with python. Proceedings of the 9th Python in Science Conference. In: van der Walt, S , Millman, J. , eds. Vol 57. SciPy;2020:92–96.

[32] Robin X , Turck N , Hainard A , Tiberti N , Lisacek F , Sanchez J‐C , Müller M . pROC: an open‐source package for R and S+ to analyze and compare ROC curves. BMC Bioinformatics. 2011;12:77. doi: 10.1186/1471-2105-12-77 21414208 PMC3068975

[33] Venkatraman ES , Begg CB . A distribution‐free procedure for comparing receiver operating characteristic curves from a paired experiment. Biometrika. 1996;83:835–848. doi: 10.1093/biomet/83.4.835

[34] Warnica W , Al‐Arnawoot A , Stanimirovic A , Thavendiranathan P , Wald RM , Pakkal M , Karur GR , Wintersperger BJ , Rac V , Hanneman K . Clinical impact of cardiac MRI T1 and T2 parametric mapping in patients with suspected cardiomyopathy. Radiology. 2022;305:319–326. doi: 10.1148/radiol.220067 35787201

[35] Ferreira VM , Schulz‐Menger J , Holmvang G , Kramer CM , Carbone I , Sechtem U , Kindermann I , Gutberlet M , Cooper LT , Liu P , et al. Cardiovascular magnetic resonance in nonischemic myocardial inflammation: expert recommendations. J Am Coll Cardiol. 2018;72:3158–3176. doi: 10.1016/j.jacc.2018.09.072 30545455

[36] Lurz JA , Luecke C , Lang D , Besler C , Rommel K‐P , Klingel K , Kandolf R , Adams V , Schöne K , Hindricks G , et al. CMR‐derived extracellular volume fraction as a marker for myocardial fibrosis: the importance of coexisting myocardial inflammation. J Am Coll Cardiol Img. 2018;11:38–45. doi: 10.1016/j.jcmg.2017.01.025 28412435

[37] Everett RJ , Treibel TA , Fukui M , Lee H , Rigolli M , Singh A , Bijsterveld P , Tastet L , Musa TA , Dobson L , et al. Extracellular myocardial volume in patients with aortic stenosis. J Am Coll Cardiol. 2020;75:304–316. doi: 10.1016/j.jacc.2019.11.032 31976869 PMC6985897

[38] Shang Y , Zhang X , Zhou X , Wang J . Extracellular volume fraction measurements derived from the longitudinal relaxation of blood‐based synthetic hematocrit may lead to clinical errors in 3 T cardiovascular magnetic resonance. J Cardiovasc Magn Reson. 2018;20:56. doi: 10.1186/s12968-018-0475-6 30089499 PMC6083590

[39] Sung TL , Lee HJ . Image‐to‐image translation using identical‐pair adversarial networks. Appl Sci. 2019;9:2668. doi: 10.3390/app9132668

[40] Lei K , Mardani M , Pauly JM , Vasanawala SS . Wasserstein GANs for MR imaging: from paired to unpaired training. IEEE Trans Med Imaging. 2021;40:105–115. doi: 10.1109/TMI.2020.3022968 32915728 PMC7797774

[41] Esser P , Kulal S , Blattmann A , Entezari R , Müller J , Saini H , Levi Y , Lorenz D , Sauer A , Boesel F , et al. Scaling Rectified Flow Transformers for High‐Resolution Image Synthesis. arXiv. 2024:2403.03206.

[42] Dosovitskiy A , Beyer L , Kolesnikov A , Weissenborn D , Zhai X , Unterthiner T , Dehghani M , Minderer M , Heigold G , Gelly S , et al. An Image Is Worth 16x16 Words: transformers for Image Recognition at Scale. arXiv. 2020:2010.11929.

