# Supplementary material for: Deep Learning Virtual Contrast‐Enhanced T1 Mapping for Contrast‐Free Myocardial Extracellular Volume Assessment
Source: J Am Heart Assoc. 2024 Sep 30;13(19):e035599. doi: 10.1161/JAHA.124.035599 (PMC11681454; doi:10.1161/JAHA.124.035599)
Supplement: Supplementary file 1 — Data S1–S4 Figures S1–S4 [file JAH3-13-e035599-s001.pdf]

Supplemental Material

Supplemental Methods

Data S1: Detailed information on the data pre-processing

The native and contract-enhanced (CE) T1 map pairs were pre-processed prior to training the deep learning method:

i) **Myocardial segmentations** were conducted by a standard U-Net model implemented in MONAI v0.9.0 for both native and CE T1 maps. To train this U-Net model, the myocardium in native and CE T1 maps was manually segmented by L.M.B. in 100 separate patients.

ii) **Rigid registration** was performed on all cropped image pairs using SimpleITK v2.2.0, while considering only image areas within the segmented myocardium and a 1cm border in the registration optimization. Pairs were excluded if visual inspection revealed that rigid registration failed due to obvious dissimilar orientation and/or cardiac phase between native and CE T1 maps.

iii) **Image cropping** of registered T1 map pairs was conducted based on the myocardial segmentation. The cropped areas were centrally written into two 128x128 matrices.

iv) **T1 values normalization** was done by clipping the native T1 map values to the range 0 to 2000 ms and the CE T1 map values to 0 to 800 ms and then linearly transforming them to a range 0-1.

Data S2: Detailed information on the deep learning training

Training cases with amyloidosis were oversampled (i.e., drawn two times per epoch) to increase the representation of amyloidosis during training. AdamW optimizer with beta (0.5, 0.999), a learning rate of 0.0002 that is decreased with factor 0.5 ten times during training, weight decay of 0.1 and a batch size of 64 with gradient accumulation over 6 batches was used during 20000 epochs of training. Training time for one of five validation splits was 32 hours on a Nvidia RTX 3090 GPU.

Data S3: Detailed information of the random forest regressor training

We compared the GAN-based approach to a random forest regression (RFR). As with the GAN, we developed the RFR on the training set (without hematocrit) to predict mean vCE T1 values of myocardium and of the blood pool based on the mean native T1 values of myocardium and the blood pool. In contrast to the GAN we and additionally included age and gender of the patient as input variables for the RFR. RFR was implemented using Scikit-learn v1.1.2 using default paramerters that were: (criterion = 'squared_error', max_depth = None, n_estimators = 100, min_samples_split = 2, min_samples_leaf = 1, min_weight_fraction_leaf = 0.0, max_features = 1.0, max_leaf_nodes = None, min_impurity_decrease = 0.0, bootstrap = True, oob_score = False, n_jobs = None, random_state = None, verbose = 0, warm_start = False, ccp_alpha = 0.0, max_samples = None, monotonic_cst = None).

Data S4: Detailed information on the region of interest used for extracellular volume determination

Extracellular volume (ECV) was calculated with the following formula using native and contrast-enhanced (CE) T1 values within a myocardial (myo) region of interest (ROI) and a blood ROI:

$$\mathrm{ECV} = \frac{\Delta\mathrm{R1\ myo}}{\Delta\mathrm{R1\ blood}} * (1 - \mathrm{haematocrit})$$

$$\Delta\mathrm{R1\ myo} = \frac{1}{\mathrm{native\ T1\ myo}} - \frac{1}{\mathrm{CE\ T1\ myo}} \qquad \Delta\mathrm{R1\ blood} = \frac{1}{\mathrm{native\ T1\ blood}} - \frac{1}{\mathrm{CE\ T1\ blood}}$$

The myo and blood ROI were determined from the U-Net based segmentations conducted during pre-processing. To avoid partial volume effects, the myo segmentation was morphologically eroded. To identify a blood ROI, the segment surrounded by the myocardial segmentation was post-processed by applying otsu-threshold to a median filtered version of the image (kernel size of 5x5), followed by extraction of the largest contiguous mask and erosion. For calculation of vECV the ROIs of the native T1 map was also applied to the vCE T1 map that were generated from the native T1 map. These ROIs used to calculate the ECV and vECV were visually inspected and corrected if necessary.

Figure S1: Example T1 maps that were excluded due to bad map quality

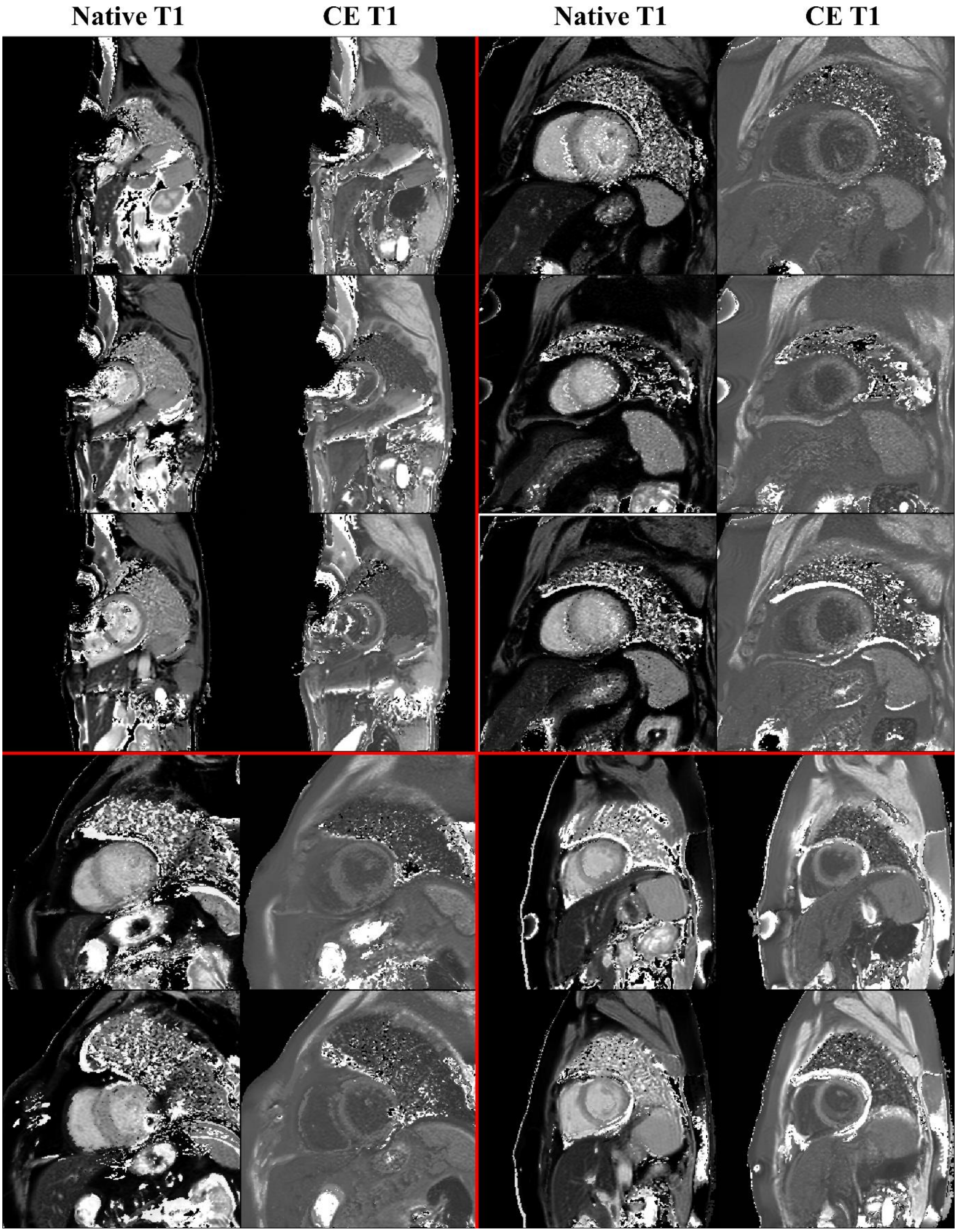

Examples of T1 maps that were excluded due to bad T1 map quality from training and evaluating the machine-learning methods.

Figure S2: Additional example T1 maps that were excluded due to bad map quality

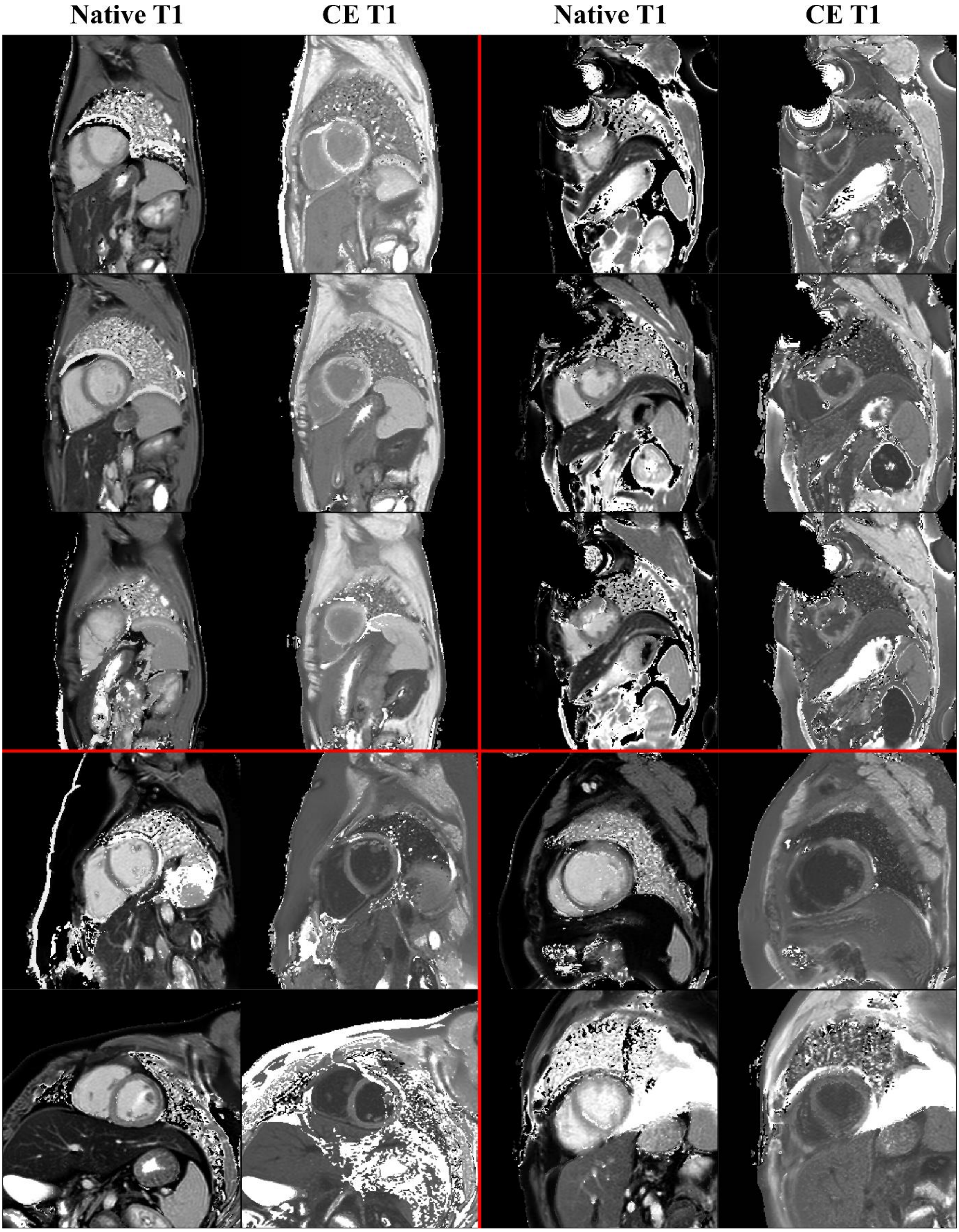

Examples of T1 maps that were excluded due to bad T1 map quality from training and evaluating the machine-learning methods.

Figure S3: Examples for excluded native and CE T1 map pairs with failed rigid registration due to dissimilar slice T1 map location or myocardial contraction phase.

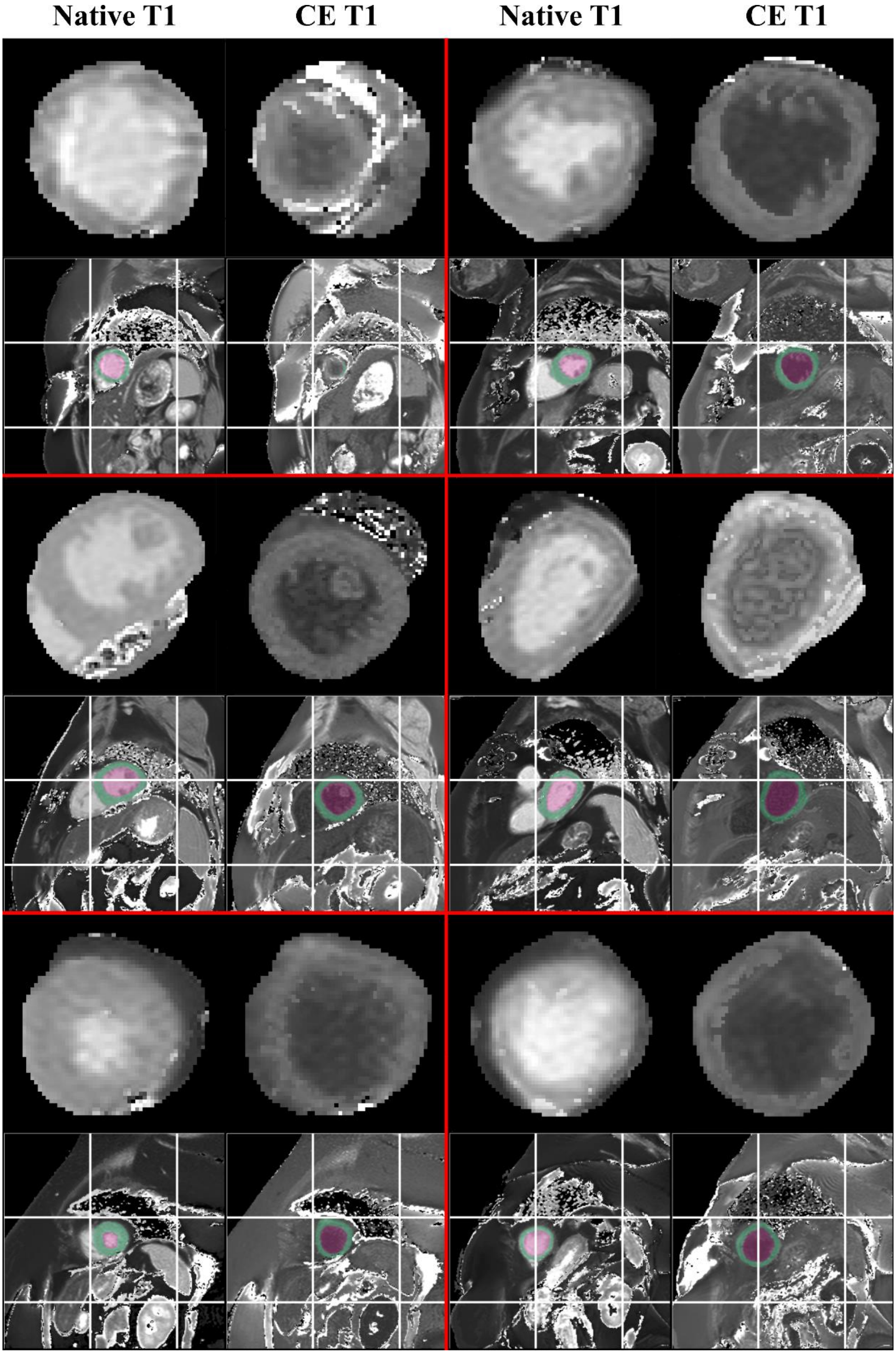

Examples of native and contrast-enhanced (CE) T1 maps for which rigid registration and conventional extracellular volume calculation was infeasible.

Figure S4: Additional examples for excluded native and CE T1 map pairs with failed rigid registration due to dissimilar slice T1 map location or myocardial contraction phase.

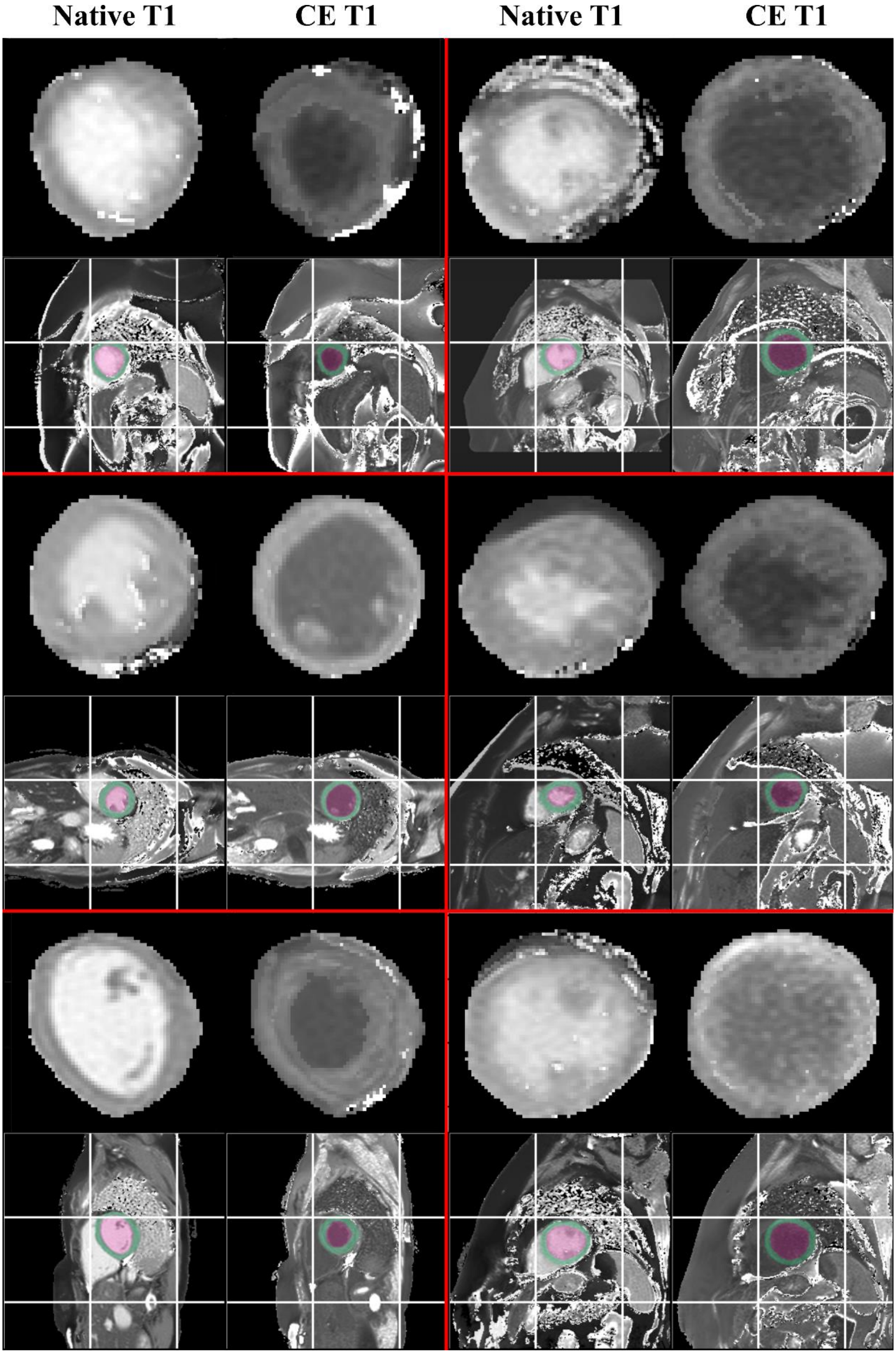

Examples of native and contrast-enhanced (CE) T1 maps for which rigid registration and conventional extracellular volume calculation was infeasible.
